# Destroying the Nerve and Fang Filling

**Published:** 1859-02

**Authors:** Joseph Mansfield

**Affiliations:** Niles, Mich.


### DESTROYING THE NERVE AND FANG FILLING.

BY JOSEPH MANSFIELD, OF NILES, MICH.

Read before the Michigan State Dental Society, at its 4th Annual Meeting, held in Detroit, Jan-11th, 1859.

I always destroy the nerve when exposed. I have tried capping; it is with but poor success. I lose one out of four in capping.

Take arsenic, one part, morphine or tannin, one part, make it into a thin paste with creosote, then take a piece of small cotton twine, cut it into very short pieces, put it in your paste, cork it up tight and let it remain until it becomes well steeped in the paste; take out your cork, let it evaporate until it becomes nearly dry before using. One piece of the twine is sufficient to destroy the nerve. (I do this to prevent getting it on the gum, and it is much easier to get into the cavity.) I direct my patients to be sure and come the next morning, and have it taken out—(if at a distance, to take it out themselves.) If the patient is young, from 12 to 16, don't let it remain over 8 or 10 hours. I now partially cleanse the cavity. If not too sensitive, I prick the nerve so as to cause it to bleed, (I think this quite essential,) then I put in a small pledget of cotton saturated with creosote, let it remain from 2 to 4 days, and repeat it from time to time until the soreness has left, then clean the nerve and crown cavity thoroughly. I usually take a small drill and drill the fang as far as I can, then introduce a small piece of cotton saturated with creosote into the fang, stop the cavity with wax or cotton, let it remain from 2 to 3 days, if no soreness, then fill the fang and crown cavity. If there is soreness repeat the creosote.

I have on my note book 17 teeth treated in this way from the 21st of January to the last of February, which I have seen or heard from within a few weeks, and are doing well and perfectly satisfactory to the patient.

I filled 10 teeth during the same time, where the nerve was dead; some discharged through the fang and a part through the gum, which are all doing well. These I treated with caustic, chloride of zinc, and creosote. In the first place I take a small piece of caustic, crowd it as far as possible into the fang, stop the cavity with cotton; if the discharge is through the gum, I take a stout gum lancet and cut through the gum to the fang of the tooth; at the orifice of the discharge put in a piece of caustic. I order my patient to come the next day, then partially clean the fang and crown cavity; after this I use creosote mostly, sometimes cloride zinc. Treat in this way from two to four weeks, as the case may require, then introduce cotton saturated with strong creosote far up into the fang. Then fill with temporary filling, (I have used the New York Tooth Manufacturing-Co's polishing sticks, made of gutta percha and silex,) let it re main in from two to six weeks, then fill the fang. If you think filling the crown at the same time will be likely to produce inflammation, defer it for a few days.

I do not lose one front or bicuspids tooth in twenty with this treatment, in destroying nerve or where it has died from exposure.

You can not treat molar teeth with the same success from the fact that it is more difficult to thoroughly cleanse and fill the fangs.

